# Vitamin D and Risk of Incident Type 2 Diabetes in Older Adults: An Updated Systematic Review and Meta-Analysis

**DOI:** 10.3390/nu16111561

**Published:** 2024-05-22

**Authors:** Ligia J. Dominguez, Nicola Veronese, Eliana Marrone, Carla Di Palermo, Candela Iommi, Rosaria Ruggirello, Carla Caffarelli, Stefano Gonnelli, Mario Barbagallo

**Affiliations:** 1 Department of Medicine and Surgery, “Kore” University of Enna, 94100 Enna, Italy; 2 Geriatric Unit, Department of Medicine, University of Palermo, 90127 Palermo, Italy; nicola.veronese@unipa.it (N.V.); eliana94marrone27@gmail.com (E.M.); carladipalermo@gmail.com (C.D.P.); candelaiommi@gmail.com (C.I.); rosariaruggirello34@gmail.com (R.R.); mario.barbagallo@unipa.it (M.B.); 3 Section of Internal Medicine, Department of Medicine, Surgery and Neuroscience, University of Siena, 53100 Siena, Italy; carla.caffarelli@unisi.it (C.C.); stefano.gonnelli@unisi.it (S.G.)

**Keywords:** vitamin D, diabetes, hypovitaminosis D, aging, elderly, older adults, meta-analysis

## Abstract

Vitamin D deficiency is very common worldwide, particularly in old age, when people are at the highest risk of the negative adverse consequences of hypovitaminosis D. Additionally to the recognized functions in the regulation of calcium absorption, bone remodeling, and bone growth, vitamin D plays a key role as a hormone, which is supported by various enzymatic, physiological, metabolic, and pathophysiological processes related to various human organs and systems. Accruing evidence supports that vitamin D plays a key role in pancreatic islet dysfunction and insulin resistance in type 2 diabetes. From an epidemiological viewpoint, numerous studies suggest that the growing incidence of type 2 diabetes in humans may be linked to the global trend of prevalent vitamin D insufficiency. In the past, this association has raised discussions due to the equivocal results, which lately have been more convincing of the true role of vitamin D supplementation in the prevention of incident type 2 diabetes. Most meta-analyses evaluating this role have been conducted in adults or young older persons (50–60 years old), with only one focusing on older populations, even if this is the population at greater risk of both hypovitaminosis D and type 2 diabetes. Therefore, we conducted an update of the previous systematic review and meta-analysis examining whether hypovitaminosis D (low serum 25OHD levels) can predict incident diabetes in prospective longitudinal studies among older adults. We found that low 25OHD was associated with incident diabetes in older adults even after adjusting for several relevant potential confounders, confirming and updating the results of the only previous meta-analysis conducted in 2017.

## 1. Introduction

According to the International Diabetes Federation (IDF) Diabetes Atlas, the global diabetes prevalence in people 20–79 years old in 2021 was estimated to be 10.5% (536.6 million people), with a prospective 12.2% increase (783.2 million) by 2045. The number of people diagnosed with type 2 diabetes has increased fivefold in the past 25 years. As in previous estimates, diabetes prevalence was highest in older adults, particularly in those aged 75–79 years. Due to its association with relevant complications, global diabetes-related health expenditures were estimated at 966 billion USD in 2021 and are projected to reach 1054 billion USD by 2045 [1]. These current numbers and projections make diabetes one of the most devastating diseases of the 21st century [2].

Long-lasting diabetes can lead to a wide range of complications such as chronic pain [3], lower limb amputations [4], increased healthcare costs [5,6,7], falls [8], and cognitive decline and dementia [9,10], among others. Even if pharmacological treatments for diabetes have recently advanced significantly, there are still no effective interventions in the prevention of this alarming disease, and governments have proven to be slow to adopt the action necessary for diabetes prevention [11].

A deficit of vitamin D is a potential reversible target for diabetes prevention in older adults. In fact, another condition for which interest has grown in recent years is hypovitaminosis D, which has been increasingly reported to be associated with several negative health outcomes, including an increasing risk of incident diabetes [12,13]. Furthermore, vitamin D deficit is extremely frequent, particularly in old age [14] as well as in patients with type 2 diabetes [15].

Several mechanisms may help explain the link between vitamin D and type 2 diabetes, including vitamin D-mediated stimulation of pancreatic insulin secretion, the association of hypovitaminosis D with metabolic syndrome and with inflammation, as well as genetic polymorphisms of vitamin D that may lead to impaired blood glucose control [16,17].

Previous observational studies showed an inverse association between 25 hydroxyvitamin D (25OHD) and the risk of incident type 2 diabetes confirmed in a meta-analysis by Song et al., including twenty-one prospective observational studies [18]. However, intervention studies have been either negative or showed only limited benefit [19,20]. A former meta-analysis of eight trials did not show any effect of supplementing vitamin D on incident diabetes or glycemic control [21]. More recently, a meta-analysis by Barbarawi et al., including nine randomized controlled trials (RCTs) (43,559 participants), reported that in patients with prediabetes, vitamin D supplementation at moderate to high doses (≥1000 IU/day) significantly reduced the risk of incident type 2 diabetes, compared with the placebo [22]. However, the significant association persisted only for those with a body mass index (BMI) lower than 30 kg/m^2^ and was not significant for those with a BMI higher than 30 kg/m^2^. Similarly, a meta-analysis by Zhang et al., including eight RCTs of patients with prediabetes (4896 participants), comparing vitamin D vs. the placebo (regardless of dose, type, route of administration, and duration) showed that overall vitamin D supplementation significantly reduced the risk of type 2 diabetes, but group analysis showed that the benefit was found in nonobese subjects but not in obese subjects [23]. Pittas et al. have recently reported the results of a meta-analysis including only RCTs specifically designed and conducted to test the effects of oral vitamin D vs. the placebo on new-onset diabetes in adults with prediabetes [24]. Among three RCTs with a low risk of bias, vitamin D reduced the risk for incident diabetes by 15% in adjusted analyses. In participants in the intervention arm who maintained a mean serum 25OHD level ≥50 ng/mL vs. 20–29 ng/mL throughout follow-up, vitamin D supplementation reduced the risk for diabetes by 76%, with a 3-year absolute risk reduction of 18.1%. Even if the results are remarkable, the authors admit that they may not be directly applicable to the general population because the studies included people with prediabetes, which was an inclusion criterion of the meta-analysis [24].

All the above-mentioned meta-analyses [18,22,23,24], which support hypovitaminosis D as a possible key determinant of incident type 2 diabetes, have been done in populations of adults or young older adults (aged 50–60 years). Most studies excluded older people, even if this is the population at greater risk for both hypovitaminosis D [14,25] and type 2 diabetes [1,26]. A previous meta-analysis, including nine prospective studies (28,258 participants), investigated whether hypovitaminosis D was associated with incident diabetes among older participants (mean age: 67.7 years; median follow-up: 7.7 years). The authors found that lower levels of 25OHD were associated with a significantly higher risk of incident diabetes compared with higher levels of 25OHD. The results remained significant even after adjustments for a median of eleven potential confounders among the available studies [27].

Given this background, we conducted an update of the previous systematic review and meta-analysis examining whether hypovitaminosis D (a low serum 25OHD level) can predict incident diabetes in prospective longitudinal studies conducted among older adults.

## 2. Materials and Methods

The present systematic review was performed following strengthening the reporting of observational studies in epidemiology [STROBE] criteria [28] and the recommendations in the preferred reporting items for systematic reviews and meta-analyses [PRISMA] statement [29].

### 2.1. Search Strategy

Four authors in couples independently searched for longitudinal studies that considered diabetes and serum 25OHD levels among older people. Major databases (PubMed, SCOPUS) were examined from 10 July 2016 until 15 October 2023, without language restrictions.

The search strategy used in PubMed was: (vitamin D OR 25-hydroxyvitamin D OR calcidiol OR calcitriol OR calcifediol) AND (insulin OR glucose OR beta-cell function OR diabet*) AND (old OR elderly OR older OR aged). An analogous search (adapted to the requirements of Scopus) was performed.

### 2.2. Eligibility Criteria and Study Selection

We considered the following requisites for articles to be eligible: (1) longitudinal prospective design; (2) assessment of serum 25OHD considering that it is currently accepted as the best indicator for vitamin D status [19]; (3) including only older people (mean age of participants ≥60 years; (4) self-reported diagnosis of diabetes during follow-up, or diagnosis through medical and/or hospital records, or at least one diagnostic criterion according to the American Diabetes Association (i.e., glycosylated hemoglobin (HbA1c) ≥6.5%, fasting plasma glucose (FPG) ≥126 mg/dL, or use of antidiabetic medications). Studies with the following characteristics were excluded: (1) having a cross-sectional design; (2) assessment of vitamin D status with methods different than serum 25OHD (e.g., dietary intake of vitamin D); (3) including only subclinical estimations of diabetes (e.g., changes in FPG). References of the included articles were hand-searched in order to identify additional, potentially pertinent publications. Abstracts from conferences were also examined, and in such cases, we contacted the corresponding authors to obtain the data enabling inclusion.

### 2.3. Data Extraction

Data extracted from the selected studies were independently recorded into a standardized Microsoft Excel spreadsheet by one author checking as well the quality of the extracted data. Any discrepancy was resolved by agreement with a third expert author. For each study, the following information was extracted: (i) characteristics of the study (e.g., demographics, sample size, country in which the study was conducted); (ii) setting of the study; (iii) number of people developing diabetes during the follow-up period; (iv) number of years of follow-up; (v) levels of serum 25OHD and methods utilized for the assessment of 25OHD; (vi) diagnostic criteria considered for the formulation of diabetes diagnosis; (vii) type and number of covariates included in the multivariable analyses. If the study included >2 categories for serum 25OHD, we considered the lowest quantile as low level and the highest quantile as the reference group.

### 2.4. Study Quality Assessment

One investigator assessed the quality of the study, which was independently checked by another one. The assessment of the study quality was performed using the Newcastle–Ottawa Scale (NOS) [30]. This scale allocates a maximum of nine points according to three quality parameters: selection, comparability, and outcome.

### 2.5. Statistical Analysis

A random effects meta-analysis was undertaken using MedCalc^®^ Statistical Software version 22.023 (MedCalc Software Ltd., Ostend, Belgium; https://www.medcalc.org; accessed on 1 March 2024) to take into account the expected heterogeneity. The primary analyses synthesize data by calculating pooled RRs and 95% CI (including the number of incident cases of diabetes in higher levels of serum 25OHD vs. lower values). Secondary analyses considered the hazard ratio/odds ratio (HR/OR) adjusted for the highest number of available covariates for each study examined together using fully adjusted RRs. Heterogeneity among the included studies was evaluated using chi-squared and I-squared statistics, assuming a *p* < 0.05 and a value ≥ 50%, respectively, indicative of a significant heterogeneity [31]. When we found significant heterogeneity, and there were ≥4 studies available, metaregression analysis was preplanned considering the potential moderators: (1) continent in which the study was performed; (2) study setting (community vs. others); (3) follow-up in years (continuous); (4) diagnostic criteria utilized in the formulation of diabetes diagnosis (self-reported vs. others); and the number of covariates; (5) visual inspection of funnel plots and the Egger bias test were used to assess publication bias [32]. We used the Duval and Tweedie nonparametric trim-and-fill method to examine the potential publication bias when ≥3 studies were available. Assuming that the effect sizes of all the studies were normally distributed around the center of a funnel plot, in the event of asymmetries, this procedure adjusts for the potential effect of unpublished (trimmed) studies [33].

## 3. Results

### 3.1. Literature Search

**Figure 1:**
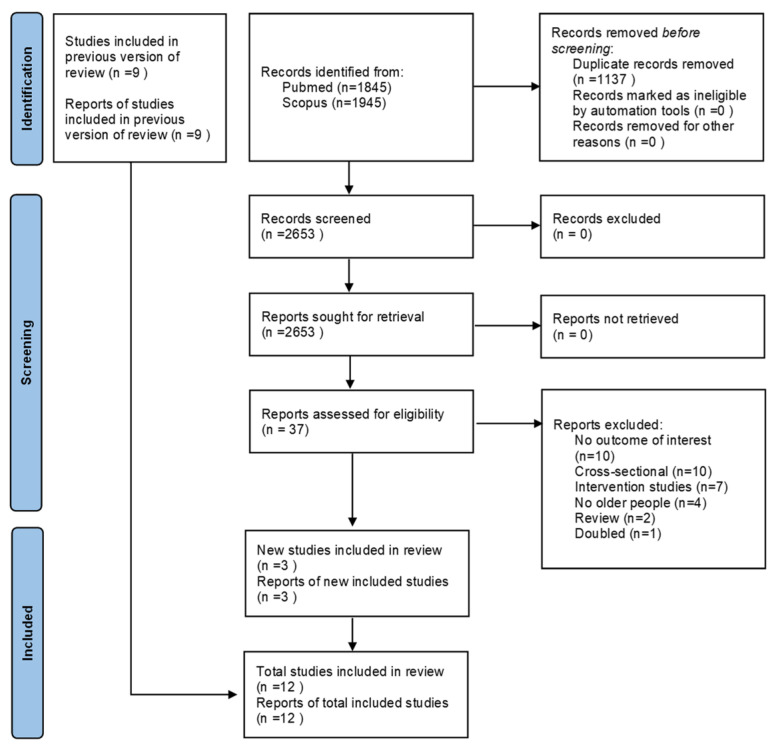
PRISMA flow-chart.

As shown in Figure 1, after the update, we identified 2653 nonduplicated, potentially eligible studies. Of them, 37 full texts were examined, and three were eligible for the update of our meta-analysis for 12 works [34,35,36,37,38,39,40,41,42,43,44,45].

### 3.2. Study and Participants Characteristics

In Table 1, the characteristics of the 12 included studies involving 40,664 older participants, followed up for a median period of 7.3 (range: 2–14) years, are shown. From the total participants at baseline, 4263 participants (10.5% of the baseline population) developed diabetes. The mean age of the participants was 69.1 years, and most of them were women (66%). All the studies were carried out among community dwellers, mainly in European (n. of studies = 6) and North American (n. of studies = 5) populations. As shown in Table 1, most of the studies used the radioimmunoassay (RIA) method for assessing 25OHD levels. In none of the studies included, did the authors report baseline vitamin D supplementation by the future risk of diabetes. Regarding the diagnosis of diabetes, two studies used only self-reported information. Other studies used self-reported information, followed by a final specialist adjudication in four studies, with a value of FPG over 126 mg/dL in another one and with the use of antidiabetic medications in a third study. Two studies used a value of HbA1c over 6.5% for the diagnosis of type 2 diabetes, one only a value of FPG over 126 mg/dL, and one study considered all the criteria suggested by the American Diabetes Association.

The median NOS values (median = 7; range: 5–9) indicate that the quality of the studies was generally of moderate quality (Table 1).

### 3.3. Unadjusted Findings Concerning Lower 25OHD Levels and Diabetes

**Figure 2:**
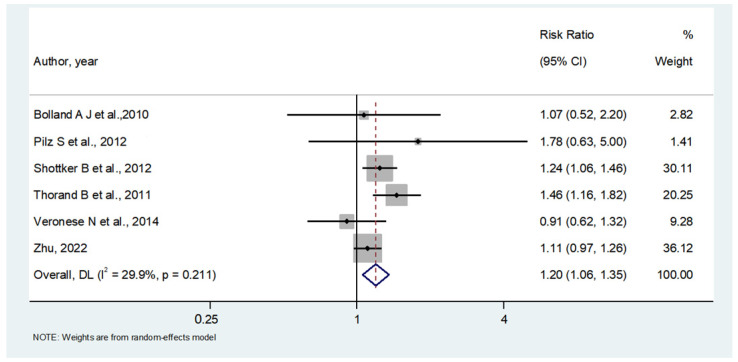
Association between lower 25 hydroxyvitamin D (25OHD) levels at baseline and incident diabetes [34,37,40,43,44,45].

**Figure 3:**
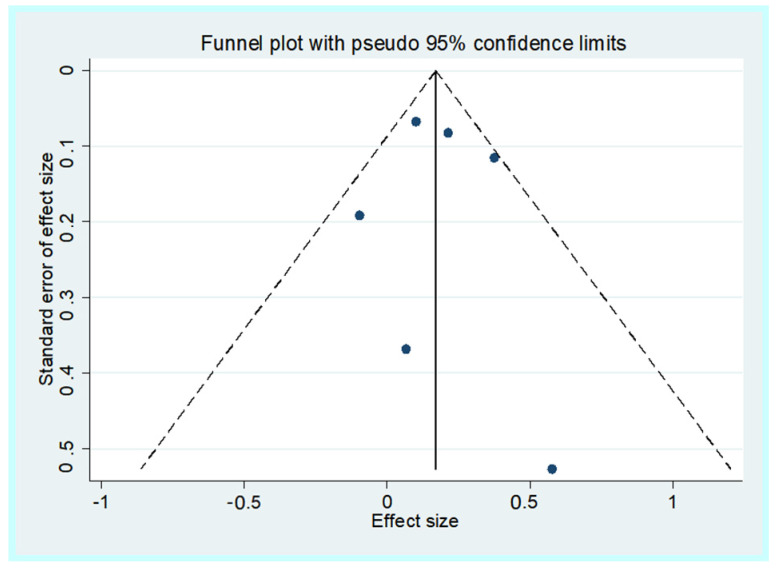
Funnel plot of the unadjusted associations.

Figure 2 shows the results of the meta-analysis, including 960 older participants with lower 25OHD (from a total of 6717 participants; 14.3%) and 1000 participants with higher 25OHD (from a total of 9207 participants; 10.9%) with a diagnosis of diabetes during the follow-up period. Lower baseline 25OHD levels were associated with a higher risk of incident diabetes vs. older people with higher 25OHD levels: this analysis included a total of 15,924 older participants and reported an RR = 1.20; 95% CI: 1.06–1.35; *p* = 0.001; I^2^ = 29.9%). No publication bias was found (Egger’s test = 0.26; *p* = 0.85) (Figure 3), and the trim and fill analysis did not modify our results.

### 3.4. Adjusted Analyses Concerning Lower 25OHD Levels and Diabetes

**Figure 4:**
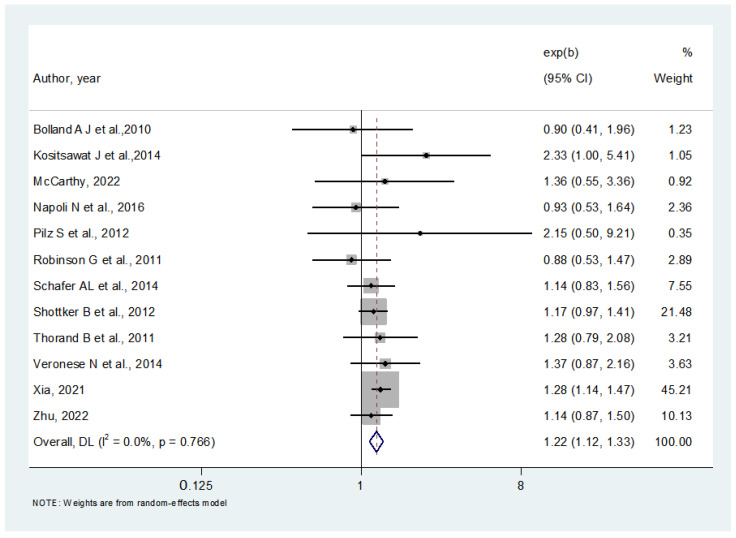
Association between lower 25-hydroxyvitamin D (25OHD) levels at baseline and incident diabetes, after adjusting for potential confounders [34,35,36,37,38,39,40,41,42,43,44,45].

**Figure 5:**
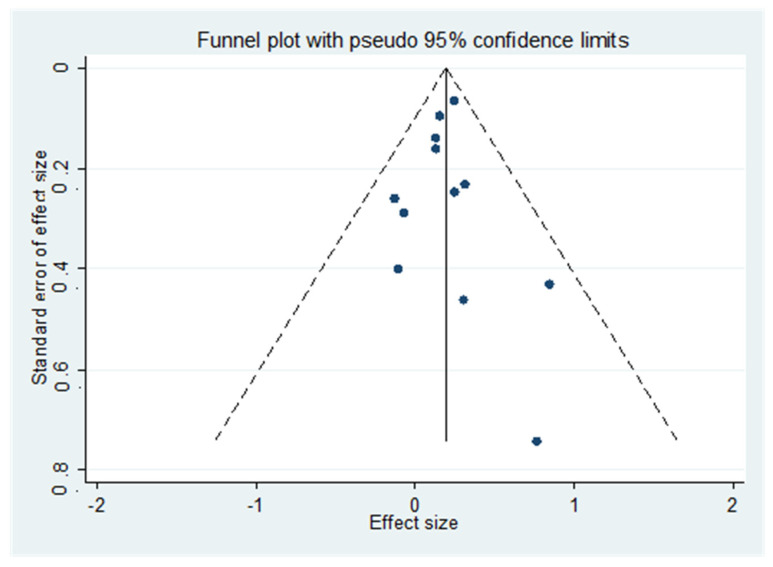
Funnel plot of the adjusted associations.

Figure 4 shows the association between lower baseline serum 25OHD levels and incident diabetes at follow-up after adjusting for potential confounders. The median number of adjustments in the multivariable analyses was 11 (range: 4–17), with all the studies including adiposity estimates, as fully detailed in Table 1. In the 12 studies included, we observed that lower baseline 25OHD levels increased the risk of developing diabetes by 19% (HR = 1.22; 95% CI: 1.12–1.33; *p* < 0.0001; I^2^ = 0%). We did not observe publication bias (Egger’s test = −0.34; *p* = 0.87) (Figure 5), and the trim and fill analysis did not affect our original findings.

### 3.5. Metaregression and Sensitivity Analysis

We did not observe high heterogeneity among the outcomes included in our analyses; therefore, metaregression analyses were not performed. However, since serum 25OHD concentrations may change during the time [46], we did run a metaregression analysis overall, showing that a longer follow-up did not moderate for unadjusted (beta = 0.01; *p* = 0.67; R2 = 0%) or adjusted (beta = 0.03; *p* = 0.85; R2 = 0%) data.

**Figure 6:**
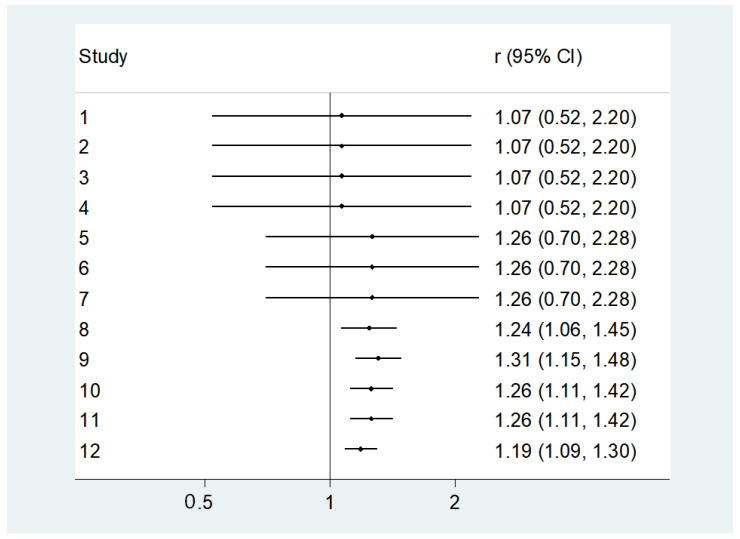
Cumulative analysis for unadjusted data.

**Figure 7:**
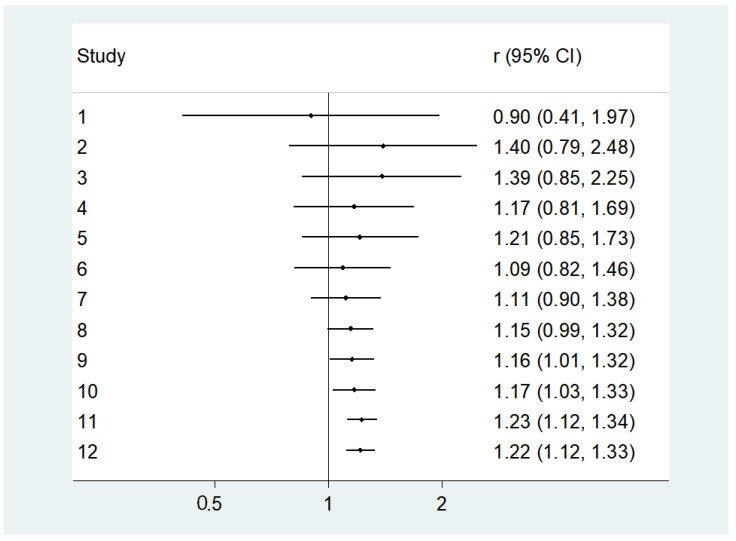
Cumulative analysis for adjusted data.

We also did a cumulative analysis, removing one study at each time, but the results were unchanged, as shown in Figure 6 and Figure 7.

## 4. Discussion

In the present meta-analysis, including 12 longitudinal prospective studies and 40,664 older participants, we found that low baseline levels of circulating 25OHD (hypovitaminosis D) were significantly associated with incident diabetes at follow-up (mean of 7.3 years), after adjusting for several potentially relevant confounders.

Noteworthy, none of the prospective studies included reported a significant relationship between baseline hypovitaminosis D and incident diabetes in the multivariable-adjusted analyses. Conversely, when the data were combined in the meta-analysis, including a median of 11 potential confounders, the presence of hypovitaminosis D at baseline was significantly associated with an increased risk of incident diabetes at follow-up by 19%. Our findings suggest that hypovitaminosis D may be a potentially reversible risk factor for the development of diabetes in older adults, who are at the highest risk of both hypovitaminosis D and type 2 diabetes and of their disabling consequences.

As mentioned above, previous studies including adults (aged 50–60 years) did not report unambiguous results regarding the relationship between vitamin D deficiency and type 2 diabetes. Although observational studies showed an inverse association between 25OHD and the risk of incident type 2 diabetes [18], intervention studies were negative or showed limited benefit [19,20,21]. More recent meta-analyses have reported that vitamin D supplementation significantly reduced the risk of incident type 2 diabetes in patients with prediabetes either considering a moderate to high dose (≥1000 IU/day) [22] or regardless of type, dose, duration, and route of administration [23]. Interestingly, in both of these meta-analyses, the significant association persisted only for nonobese participants and was not significant for obese participants. This is particularly relevant for older adults since, in this population, adiposity appears to be an important factor determining low 25OHD levels [47], which may help explain the results of some studies reporting no association between vitamin D and diabetes in older people included in our meta-analysis. These studies may have been negative or neutral when considered in isolation, but by increasing the statistical power by means of the meta-analysis, the final results showed a significant inverse association between low 25OHD levels and incident diabetes in older adults.

The most recent meta-analysis by Pittas et al. [24] with a very rigorous and well-conducted design showed that supplementation with vitamin D reduced the risk for incident diabetes by 15% in adjusted analyses, reaching a reduction of 76% in participants maintaining 25-OHD levels ≥50 ng/mL vs. 20–29 ng/mL during follow-up. These results are outstanding; however, the meta-analysis included a population of adults or not very old people rather than older adults who are at greatest risk of both, hypovitaminosis D [14,25] and type 2 diabetes [1,26].

An observational study based on data from the vitamin D and type 2 diabetes (D2d) study, examined whether intratrial vitamin D exposure affected the risk of incident diabetes in adults with prediabetes. The authors found that the HR for diabetes among participants treated with vitamin D who maintained intratrial 25OHD levels of 100–124 and ≥125 nmol/L were 0.48 (0.29–0.80) and 0.29 (0.17–0.50), respectively, compared with those who maintained a level of 50–74 nmol/L, suggesting that vitamin D supplementation should maintain 25OHD serum level ≥100 nmol/L (≥40 ng/mL) in order to reduce the risk of diabetes in adults with prediabetes [48].

In a cohort study of 903 adults with a mean age of 74 years, higher 25OHD concentrations (>30 ng/mL) were associated with significantly lower risk for diabetes with an inverse dose-response gradient: each 10 ng/mL higher 25OHD concentration was associated with a 36% lower risk of developing diabetes [49]. This study was not included in the analysis due to a too-long follow-up time (12 years) since the 25OHD measurement.

Another crucial point to be considered in the interpretation of our results is the evidence that low vitamin D levels are associated with a higher risk of mortality [50,51,52]. Therefore, it is possible that older persons with low serum 25OHD levels may die before evolving into diabetes.

Several mechanisms may help explain our findings of a significant association between low vitamin D levels and an increased risk of incident diabetes, including vitamin D’s modulation of insulin secretion [16,53,54,55]; a reduction of insulin resistance with evidence of vitamin D receptor (VDR) presence in the major insulin-sensitive tissues, i.e., adipocytes [56], muscle [57], and hepatocytes [58]; improved insulin reactivity for glucose transport [59,60]; indirectly through the regulation of calcium [60,61,62,63,64] and magnesium [65,66,67] metabolism, essential for intracellular processes mediated by insulin; a reduction of chronic inflammation [67,68,69,70,71]; because most patients affected by type 2 diabetes are obese, they may have repression of the bioactivation of vitamin D by cytochrome P450 2R1 [72,73] leading to reduced production of 25OHD; and finally, vitamin D sequestration in excess adipose tissue may blunt vitamin D serum levels [14,74].

Regarding insulin secretion, beta cells express both VDR and 1 alpha-hydroxylase (Cyp27b1), which catalyzes the activation of 25OHD into 1,25(OH)_2_D, denoting the cell-intrinsic role for VDR [75]. Nevertheless, even if the relationship between vitamin D levels and islet function is significant, whether vitamin D supplementation can directly ameliorate insulin secretion in humans remains uncertain because intervention clinical trials have reported mixed results [75,76,77,78]. As regards the ubiquitous expression of VDR, and, in particular, in insulin-sensitive tissues, a study analyzing human and rodent data showed that: (1) 25OHD repletion in insulin-resistant, overweight-to-obese persons was associated with reductions in subcutaneous adipose tissue expression of pro-inflammatory and profibrotic genes, decreased collagen immunofluorescence, and improved hepatic insulin sensitivity as well as worsening trends after six months on the placebo suggesting progressive metabolic effects of 25OHD deficiency. Adipocyte-specific VDR knockout mice following a high-fat diet for 12 weeks mirrored the vitamin D-deficient humans, displaying increased adipose tissue fibrosis and inflammation and hepatic insulin resistance [56]. Because VDR detection in muscle has been controversial, a study examined differences in muscle VDR protein abundance between two mouse strains and between mice and humans. VDR protein was generally lower in some mice models and was higher in humans than in mouse muscle, suggesting that further preclinical and clinical studies are still needed to confirm possible VDR isoforms in human muscle [57]. An in vitro study on hepatocyte ischemia/reperfusion injury showed that VDR mediates hepatocyte apoptosis and proliferation not only through inflammation and immunity, as shown previously, but also through autophagy [58]. As regards glucose transport, it has been reported that 1,25(OH)_2_D improved glucose uptake via the Sirtuin 1/Insulin receptor substrate 1/glucose transporter 4 (SIRT1/IRS1/(GLUT4) axis by activation of SIRT1, phosphorylation of IRS1, and finally translocation of GLUT4 in myotubes [59]. Moreover, activation of VDR increases calcium concentrations in muscle, enhances the translocation of GLUT4, and increases glucose uptake [60]. Additionally, the pleiotropic role played by the VDR in insulin resistance may comprise: (1) increases in parathyroid hormone (PTH) induced by vitamin D, which increases insulin sensitivity by prompting higher quantities of GLUT1 and GLUT4 in vitamin D-deficient adipose tissue, muscle, and liver [79,80]; (2) suppression of the renin-angiotensin-aldosterone system activity, which damages beta cell function, inhibits peripheral insulin sensitivity [81], delays GLUT4 recruitment [82], and triggers insulin resistance [83].

Vitamin D may regulate insulin secretion and action indirectly through calcium intracellular handling. In the beta cell, 1,25(OH)_2_D leads to the depolarization of cellular membranes, the opening of calcium channels, and the elevation of intracellular calcium levels [61,64]. A possible molecular mechanism of this effect is due to the fact that 1,25(OH)_2_D activates protein kinase A and increases channel function by phosphorylating L-type voltage-dependent calcium channel-related proteins [64]. Furthermore, 1,25(OH)_2_D, through activating VDR, regulates voltage-gated calcium channel expression to improve insulin secretion [84]. An additional mechanism is that 1,25(OH)_2_D promotes the synthesis of phospholipase C and activates inositol triphosphate that releases calcium from the endoplasmic reticulum (ER) [61,85]. Vitamin D may also regulate calbindin expression [85,86], a calcium-binding protein involved in maintaining calcium concentrations. Regarding magnesium metabolism, the relationship between vitamin D and magnesium is bilateral: several steps in vitamin D metabolism (i.e., 25OHD binding and transport, liver and renal hydroxylation) depend on magnesium as a cofactor [67]. Vitamin D, in turn, plays a key role in magnesium metabolism both by stimulating intestinal magnesium absorption and preventing renal excretion [87]. Thus, it appears that the deficit of each of these two compounds feeds the deficit of the other, leading to a perverse cycle with further worsening of both deficits. The combined deficit of vitamin D and magnesium may lead to clinically relevant outcomes, including not only a higher risk of fragility fractures [88] but also altered insulin secretion and action contributing to an increased risk of incident type 2 diabetes [65,66,89].

Substantial evidence supports the role of vitamin D as an immunomodulatory hormone with notable biological effects on the innate and adaptive immune systems [12]. Vitamin D or overexpression of VDR were reported to suppress cytokine-induced pro-inflammatory responses and apoptosis in beta cell lines and islets [68,69,70], probably due to the direct suppression of nuclear factor kappa-light-chain-enhancer of activated B cells (NF-kB) activation by VDR. Besides its anti-inflammatory role, vitamin D is also an active suppressor of ER stress and islet amyloid polypeptide-induced beta cell dysfunction [68]. Vitamin D downregulates essential ER stress players in monocytes, the liver, and islets [71].

Finally, the sequestration of vitamin D inside excess adipose tissue decreases 25OHD serum levels [14,74]. There is compelling evidence of the association of an elevated body weight with low circulating 25OHD levels. Vitamin D may impact gene expression by the modulation of inflammation and adipose tissue metabolism in vitro; nevertheless, the exact metabolism of vitamin D in adipose tissue is unknown at present. White adipose tissue expresses VDR and hydroxylase enzymes, but the distribution and concentrations of the generated vitamin D compounds in adipose tissue are largely unidentified. Further options have been proposed in order to measure vitamin D compounds in adipose tissue accurately by using liquid mass spectrometry [74].

Our study has some strengths, such as being one of the few meta-analyses systematically investigating the relationship between vitamin D and incident diabetes in older adults, two relevant aspects of old age. We can also include among the strengths of our study the large number of participants included, the high number of covariates used in the adjusted analyses, a long follow-up period, particularly long considering that older adults have a lower life expectancy, and the low heterogeneity found in almost all of the outcomes included. Nevertheless, the results of the present meta-analysis should be considered taking into account its limitations as well. First, the observational nature of the included studies does not allow us to clarify causative effects and potential pathways responsible for the association of hypovitaminosis D with diabetes. Second, the mean age of the participants included was about 69.1 years. There are currently no studies in the literature on old-old people. Future studies focused on very old people, who are at the highest risk of hypovitaminosis D, are warranted to better understand this association in a vulnerable population. Third, there were a limited number of studies considering each gender; hence, we could not run analyses separated by gender, even if both serum 25OHD and diabetes are generally dissimilar in men and women. Lastly, most of the included studies measured serum 25OHD with RIA, which seems not to be the best method to measure serum 25OHD instead of chemiluminescence [90].

## 5. Conclusions

The present meta-analysis suggests that hypovitaminosis D is associated with incident diabetes in older adults even after adjusting for several relevant potential confounders, confirming and updating the results of the only previous meta-analysis conducted in older adults in 2017 [27]. This confirms the increasing evidence showing that vitamin D exerts multiple effects beyond bone and calcium metabolism. Since hypovitaminosis D is among the most frequent conditions in old age and the available RCTs investigating the role of vitamin D supplementation in the prevention of type 2 diabetes have been conducted in adults or young older adults, future larger scale well-designed studies are needed to confirm these positive results in old-old populations.

## Figures and Tables

**Table 1:** Description of the included studies characteristics.

Author, Year	Country	Sample Size	N of Subjects with Diabetes at Follow-Up	Mean Age	Percentage of Females	Mean Serum 25OHD Levels	Follow-Up (Years)	Methods of Measurement of Serum 25OHD	Diagnostic Criteria for Diabetes	Type of Covariates	Number of Covariates	NOS
Bolland A J et al., 2010 [37]	New Zealand	1472	29	74	100	50.9	5	RIA (DiaSorin, Stillwater, MN, USA)	Self-reported with a final adjudication by a specialist	Treatment allocation, age, body weight, smoking status.	4	5
Kositsawat J et al., 2014 [38]	USA	2193	477	74.6	52	65.8	2	RIA (DiaSorin, Stillwater, MN, USA)	Abnormal HbA1c (≥6.5%)	Age, sex, recruitment site, BMI, physical activity, vitamin D supplementation, multivitaminic use, season, PTH, race, diabetes status at follow-up.	11	5
McCarthy, 2022 [35]	Ireland	3373	110	62.9	53.5	57.1	4	Liquid chromatography-mass spectrometry	Self-reported doctor diagnosis of diabetes, use of diabetes medications, or an HbA1c level ≥48 mmol/mol	age, sex, educational status attained, body mass index, smoking history, physical activity, use of statins, and the season	8	7
Napoli N et al., 2016 [39]	USA	1939	139	73.3	0	27.0	6.4	Liquid chromatography tandem mass spectrometry (Thermo Fisher Scientific, Franklin, MA, USA)	FPG ≥ 126 mg/dL, self-reported	Age, site, race, season, BMI, calcium intake	6	7
Pilz S et al., 2012 [40]	The Netherlands	351	45	67.8	61	56.7	7.5	RIA (DiaSorin, Stillwater, MN, USA)	FPG ≥ 126 mg/dL F	Fasting glucose, age, sex, BMI, HDL, TG, hypertension, physical activity level, season, PTH, eGFR	11	7
Robinson G et al., 2011 [41]	USA	5140	317	66	100		7.3	DiaSorin Liaison Chemiluminescence Method (DiaSorin, Stillwater, MN)	Self-reported, drugs	Age, ethnicity, latitude, season, WHI study indicators, BMI, hypertension, fiber intake, magnesium intake, physical activity level	10	7
Schafer AL et al., 2014 [42]	USA	5463	320	76.5	100	57.4	8.6	Liquid chromatography–tandem mass spectrometry (Thermo Fisher Scientific, Franklin, MA, USA)	Self-reported	Age, clinic site, BMI, hypertension, self-reported health	5	6
Shottker B et al., 2012 [43]	Germany	7791	829	62	58	46.1	8	RIA (DiaSorin, Stillwater, MN, USA)	HbA1c ≥ 6.5%	Age, sex, season, multivitamin supplementation, frequent fish consumption, BMI, HbA1c, family history of diabetes, education, physical activity, smoking, hypertension, renal dysfunction, CRP, fasting TG	15	8
Thorand B et al., 2011 [44]	Germany	1683	416	>65	47		11	Enzyme immunoassay IDA, Frankfurt, Germany	Self- reported	Age, sex, survey, season, BMI, smoking, alcohol use, physical activity, systolic BP, total cholesterol/HDL, family history of diabetes, CRP, IL6, ICAM-1, IFN-alpha Inducible protein, markers of inflammation, waist-to-hip ratio.	17	6
Veronese N et al., 2014 [45]	Italy	2227	291	76.1	59	80.1	4.4	RIA (DiaSorin, Stillwater, MN, USA)	FPG ≥ 126 mg/dL or HbA1c ≥ 6.5% or use of medications	Age, gender, waist, hypertension, education, monthly income, smoking, eGFR, FPG, HbA1c, total cholesterol	11	8
Xia, 2021 [36]	USA	4191	453	65	100	52.0	12	Enzyme immunoassay with a competitive binding technique from Immunodiagnostic Systems Inc. (Fountain Hills, AZ, USA).	self-report of a new physician diagnosis of diabetes treated with oral drugs or insulin shots during study follow-up,	Age, clinical center, race/ethnicity, BMI, family history of diabetes, educational levels, alcohol intake, physical activity levels, cigarette smoking status, postmenopausal hormone therapy use, and season	11	7
Zhu, 2022 [34]	Germany	4841	837	61.9	57.5	45.0	14	RIA (DiaSorin, Stillwater, MN, USA)	General practitioner-confirmed patient self-reports	Age; sex; education; smoking and drinking status; vegetable, fruit, and fish consumption; regular intake of multivitamin supplements; body mass index; HbA1c; total cholesterol; high-density lipoprotein cholesterol; triglycerides; C-reactive protein; systolic blood pressure; estimated glomerular filtration rate; family history of diabetes; history of cardiovascular diseases and cancer; antihypertensive medication; lipid-lowering medication; and season of blood draw.	23	7

Abbreviations: 25OHD = 25-Hydroxyvitamin D, RIA = radioimmunoassay, HbA1c = glycated hemoglobin, BMI = body mass index, PTH = parathyroid hormone, FPG = fasting plasma glucose, HDL = high-density lipoproteins, TG = triglyceride, eGFR = glomerular filtration rate, CRP = C-reactive protein, Systolic BP = systolic blood pressure (BP), IL6 = Interleukin 6, ICAM 1 = Intercellular Adhesion Molecule 1, IFN-alpha = interferons-alpha.
